# Contributions of circadian tendencies and behavioral problems to sleep onset problems of children with ADHD

**DOI:** 10.1186/1471-244X-12-212

**Published:** 2012-11-28

**Authors:** Reut Gruber, Laura Fontil, Lana Bergmame, Sabrina T Wiebe, Rhonda Amsel, Sonia Frenette, Julie Carrier

**Affiliations:** 1 McGill University, Montreal, QC, Canada; 2 Attention, Behavior and Sleep Lab, Douglas Mental Health University Institute, Montreal, QC, Canada; 3 Centre du Sommeil et des Rythmes Biologiques, Hôpital du Sacré-Coeur de Montréal, Montreal, QC, Canada; 4 Département de Psychologie, Université de Montréal, Montreal, QC, Canada; 5 Department of Psychiatry, McGill, University, Douglas Mental Health University Institute, 6875 LaSalle Blvd, Verdun, QC, H4H 1R3, Canada

**Keywords:** Sleep onset insomnia, Externalizing problems, Sleep problems, ADHD, Circadian tendencies, Behavioral problems

## Abstract

**Background:**

Children with attention-deficit/hyperactivity disorder (ADHD) are two to three times more likely to experience sleep problems. The purpose of this study is to determine the relative contributions of circadian preferences and behavioral problems to sleep onset problems experienced by children with ADHD and to test for a moderation effect of ADHD diagnosis on the impact of circadian preferences and externalizing problems on sleep onset problems.

**Methods:**

After initial screening, parents of children meeting inclusion criteria documented child bedtime over 4 nights, using a sleep log, and completed questionnaires regarding sleep, ADHD and demographics to assess bedtime routine prior to PSG. On the fifth night of the study, sleep was recorded via ambulatory assessment of sleep architecture in the child’s natural sleep environment employing portable polysomnography equipment. Seventy-five children (26 with ADHD and 49 controls) aged 7–11 years (mean age 8.61 years, SD 1.27 years) participated in the present study.

**Results:**

In both groups of children, externalizing problems yielded significant independent contributions to the explained variance in parental reports of bedtime resistance, whereas an evening circadian tendency contributed both to parental reports of sleep onset delay and to PSG-measured sleep-onset latency. No significant interaction effect of behavioral/circadian tendency with ADHD status was evident.

**Conclusions:**

Sleep onset problems in ADHD are related to different etiologies that might require different interventional strategies and can be distinguished using the parental reports on the CSHQ.

## Background

Attention-deficit/hyperactivity disorder (ADHD) is characterized by impulsivity/hyperactivity and inattention, with symptom onset before 7 years of age and impaired functioning in two or more environmental settings [1]. ADHD is estimated to occur in 3–7.5% of school-aged children, making it one of the most prevalent child psychiatric conditions. Moreover, 50% of children diagnosed with ADHD also suffer from impairment as young adults [2]. Parental reports indicate a 2- to 3-fold higher prevalence of sleep problems in children with ADHD compared to normal controls [3], including increased bedtime resistance [4,5], delayed sleep onset [6], frequent waking in the night [7], frequent motor movements during sleep [8], and morning/daytime fatigue [9,10]. As well, approximately one-third of medication-free children with ADHD experience chronic sleep-onset insomnia (SOI) [11].

Sleep problems presenting in a child with ADHD may stress both the child and family. Furthermore, sleep disturbances can cause excessive daytime fatigue and interfere with several aspects of an individual’s daytime functioning, including mood, attention span, and behavior [12,13], which are critical to school or work performance. Accurate diagnosis and effective management of sleep problems are key to significantly improving the quality-of-life of both children with ADHD and their family members.

Despite the clinical and scientific relevance of sleep problems to the understanding and management of ADHD, the etiologies of such sleep issues remain unclear. This is particularly important for an accurate evaluation of possible causes related to parental descriptions of sleep onset problems in children with ADHD and in choosing the most effective intervention.

Amongst the potential etiologies for sleep onset problems, both behavioral and biological explanations have been advanced within the current literature. The behavioral model posits that prolonged sleep onset and delayed bedtime in children with ADHD are a result of behavioral problems, leading to increased resistance at bedtime and difficulty in settling down in the evening. According to this model, an intervention should target the behavioral problems that lead to delayed bedtime and sleep deprivation in children with ADHD. In contrast, the circadian model suggests that children with ADHD are averse to bedtime because they are sent to bed before feeling the need to sleep. In particular, it is suggested that a delay in the endogenous circadian rhythm delays sleep and waking times [14]. Such discord may lead parents to attribute child conduct to the presence of a disruptive behavioral disorder, such as ADHD, when the true problem may be an underlying circadian disorder causing both bedtime refusal [15] and increased daytime fatigue [16]. The circadian model suggests that interventions should aim to synchronize the circadian clock of children presenting with ADHD to the environmental light–dark cycle in order to effectively treat delayed sleep onset and delayed bedtimes. The recommended interventions have included melatonin administration and light therapy to reset the circadian timing system [17]. The diverse views summarized above suggest different interventional strategies, depending on the mechanism that is at play. Thus, it is of critical importance to identify the etiology.

In addition, it is also unclear if such problems are unique to children with ADHD. It has been suggested that children with ADHD may have a deficit in arousal regulation and further factors associated with sleep or bedtime behavior might, thus, be specific to this population. However, to date, this hypothesis has not been tested. As such, the objectives of this study were two-fold: The first goal was to determine the relative contributions of circadian preferences and behavioral problems in relation to sleep onset issues, specifically – sleep onset insomnia and bedtime resistance – experienced by children with ADHD and in controls. Since inconsistencies in the detection of sleep problems using PSG and parental reports have been documented, we examined and compared both sources of information. The second objective of this study was to test for a moderation effect of diagnosis (ADHD vs. control) on the impact of circadian preferences and externalizing problems on sleep problems. We hypothesized that 1) stronger evening circadian preferences would be associated with longer SOI in both children with ADHD and controls; 2) a greater level of externalizing problems would be associated with elevated bedtime resistance in both groups; and, 3) that a stronger impact of circadian evening preferences and behavioral problems on bedtime behavior would be evident in the ADHD group compared to controls.

## Methods

Participants were recruited from elementary schools in Montreal and surrounding regions. Children with ADHD and controls were recruited by flyers and advertisements through local schools, psychologists, and centers aimed at helping children with ADHD such as the Montreal Children’s Hospital and the Douglas Mental Health University Institute’s ADHD outpatient clinic. The study was approved by the Douglas Mental Health University Institute’s Research Ethics Board. Parents signed informed consent forms and all of the children assented to participation in the study.

Data collection occurred in a quiet location within participants’ home environments.

### Participants

Eighty children were screened and invited to participate in the study. Five of these children received a total score above a predetermined cutoff score on initial screening and were subsequently excluded because of the possible confound of SDB (2) or RLS (3), leaving a final study group of 26. Hence, a total of 75 children (26 ADHD, 49 Controls), aged 7–11 years (mean age = 8.61, SD = 1.27), participated in the study.

The diagnosis of ADHD was determined by criteria from DSM-IV, using the Diagnostic Interview Schedule for Children (DISC-IV) [18]; which was administered to parents. The diagnosis was then confirmed after reviewing behavior rating scales on the Conners’ Parent Rating Scale – Revised [19]. Children with T scores >60 and a diagnosis of ADHD determined by the DISC-IV were included in the ADHD group. Health-related conditions and the use of medication was assessed using a Health Screening Form that included a detailed list of questions regarding the health status of the child and the use of any medication. Children in the control group had no medical, behavioral or psychiatric condition. Both potential ADHD or control participants were excluded from the study if they had a history of autism, Tourette’s syndrome, pervasive developmental disorder, psychosis, evidence of mental retardation (IQ < 80, as assessed by the Wechsler Intelligence Scale for Children-IV) [20]; or if they had an anxiety or depressive disorder, as these disorders may affect their sleep. Moreover, because of the well-documented association between sleep-disordered breathing (SDB), Restless Leg Syndrome (RLS), Periodic Limb Movement Disorder (PLMD) and ADHD symptoms [21,22], we screened the sample for symptoms of sleep-disordered breathing using the Pediatric Sleep Questionnaire [23] exploring SDB, snoring, and sleepiness. In addition, the Chervin and Hedger tool, which investigates leg restlessness, experience of growing pains in bed, insomnia and morning headache, was employed [21] to exclude participants with symptoms of RLS. Children scoring 0.33 or higher (*i.e.,* giving positive answers to 33% or more of the 22 questions of either scale) were excluded from the study. We subsequently excluded all children with a symptom complex suggestive of these disorders.

Seventeen (65.4%) of the children with ADHD had Combined subtype ADHD, eight (30.8%) were Inattentive subtype, and one (3.8%) was Hyperactive/Impulsive subtype. As well, 33.8% of the children with ADHD had used medication for ADHD symptoms (Ritalin (4), Concerta (4), or Strattera (1)). Eight (30.8%) of the 26 patients with ADHD received a comorbid diagnosis of oppositional defiant disorder, and 2 (7.7%) received a comorbid diagnosis of conduct disorder. None of the patients with ADHD elicited symptoms suggestive of comorbid diagnosis of depression, anxiety or any other disorder which may interfere with sleep.

### Study design

**Figure 1:**
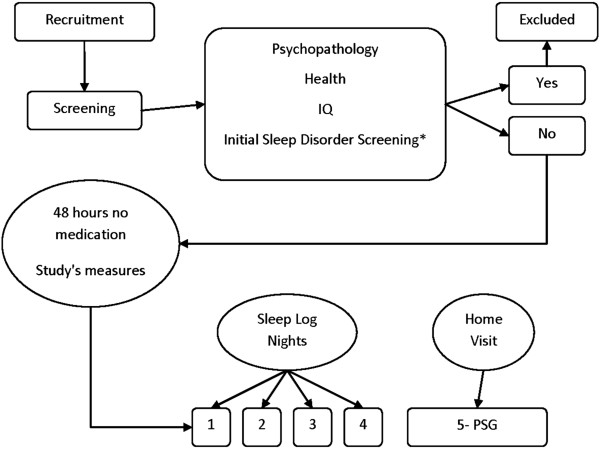
Study design.

For study design please see Figure 1. Children were first screened for eligibility to participate in the present study. During initial contact with children, over-the-phone assessment of SDB and RLS was conducted using validated questionnaires [21,24] (for details see Participants section). Children passing the over-the-phone screening then came into the laboratory and were evaluated in a quiet room.

After initial screening, eligible children were invited to participate in the study and their parents received a package that included a sleep assessment battery, standard ADHD assessment questionnaires, a demographic questionnaire, and a consent form. Parents were asked to complete and return all questionnaires. The sleep assessment battery sought to provide comprehensive baseline data on sleep-related patterns, behavior, and preferences in children with ADHD, and included the Children’s Sleep Habits Questionnaire (CSHQ) [25], the child morningness/eveningness preference scale [26], and a sleep log. As a part of the standard ADHD assessment package, parents were asked to complete both the Child Behavior Checklist [24] and the Conners’ Parent Rating Scale-Revised [19] to allow assessment of ADHD-related daytime behavior. Team evaluators were blinded to sleep assessment battery results at the time of evaluation.

Eligible participants with ADHD were asked to discontinue stimulant medication and all eligible participants were asked to avoid products containing caffeine (*e.g.,* chocolate or cola) for at least 48 hours before evaluation. The “wash-out” period of 48 hours was chosen because the half-life of the stimulant methylphenidate is, on average, 2.4 hours when either of the two dosage forms (long- or short-acting) is employed [27]. Moreover, our choice of wash-out time was consistent with the pharmacodynamic effects on behavior; the effects appear within 30 minutes, reach a peak within 1–3 hours, and dissipate within 4–6 hours [28]. Therefore, this “wash-out” period was sufficient for the purposes of the study, while allowing minimization of its impact on everyday functioning of the participants.

Parents were requested to document child bedtime over 4 nights, using a sleep log, to assess bedtime routine prior to polysomnography (PSG). On the fifth night, sleep was recorded using ambulatory assessment of sleep architecture afforded by portable PSG equipment. On the PSG night, a sleep technician arrived at each subject’s home 1.5 hours prior to habitual bedtime and hooked-up the sleep recording apparatus. Recording commenced at the child’s habitual bedtime. Sleep pattern and architecture were recorded in the natural home environment because such data affords greater ecological validity than do records logged in sleep laboratories [29,30].

## Measures

### Psychiatric and behavioral measures

#### Child behavior checklist

Overall behavioral functioning was examined using the Child Behavior Checklist (CBCL), a 113-item parental questionnaire assessing child behavior and emotional problems [24]. The CBCL is a commonly employed parental measure of youth symptoms, assessing a wide range of problems, and featuring Internalizing, Externalizing, and Total Scales. Several studies have confirmed replicability of the psychometric structure of the CBCL and high reliability, validity, and sensitivity of the scales [31,32]. In the present study, we focused on the Externalizing scale (i.e., Rule-Breaking and Aggressive Behavior categories). T-scores (converted from raw scores) provided cutoff points for criteria in the borderline/clinical range. The relevant Externalization T-score cutoff was 60, with higher scores indicating more externalizing behavior.

### Sleep assessment

#### Polysomnography

In-home polysomnography (PSG) was performed, allowing children to sleep in their natural environment. PSG recordings were performed using a digital ambulatory sleep recorder (Vitaport-3; TEMEC Instruments BV, Kerkrade, the Netherlands), measuring electroencephalography (EEG), submental electromyography (EMG), electrooculography (EOG), and finger pulse oximetry. EEG electrodes were placed bilaterally along the anteroposterior axes at locations F3, F4, C3, C4, P3, P4, O1, and O2. Sleep stages were scored visually on screen (LUNA; Stellate Systems, Montreal, Canada) using primarily central derivations (referential derivation to linked ears) according to standard AASM criteria [33]. To assess breathing two respiratory belts measuring chest and abdominal movement were used to detect hypopnea and central apneas, and pulse oximetry was used to measure oxygen saturation. The decision not to use nasal cannulae was based on the desire to not interfere with the child’s sleep, which would have impact on ecological validity. A diminution of ≥50% in chest or abdominal belt signal was considered to reflect hypopnea, whereas a complete absence of respiratory effort signal was defined as central apnea. Subjects with a hypopnea/central apnea index (AHI) >2 per hour of sleep were referred for a complete assessment of sleep related breathing disorders. Note that clinical views on the AHI values that should be used to define SDB and potential SDB differ. Previous studies on the relationship between ADHD and sleep used AHI values of 2 [34] to 5 [35] whereas some authors suggest that an AHI >1 should trigger evaluation [36]. We chose an AHI >2 for our work as it is consistent with the clinical practice of several leading Canadian sleep centers, and is at the low end of the thresholds set in previous works on the same topic. EMG leg electrodes were used to characterize leg movements during sleep. A periodic leg movement index greater than 5 per hour of sleep was considered to represent an elevated value, and these subjects were excluded from the study. Various PSG sleep measures were analyzed, but, in the present study the primary variable was sleep latency. In addition, sleep duration, the amount of time spent in each sleep stage, and sleep efficiency were calculated.

Scoring of sleep parameters was performed by certified sleep technicians blinded to all assessment and survey data.

#### Reported sleep problems

Reported sleep problems were examined using the Children’s Sleep Habits Questionnaire (CSHQ) [25], a retrospective, 33-item parent questionnaire that has been employed in several studies examining sleep behavior in young children. The CSHQ includes items exploring a number of key sleep domains and shows good validity and reliability [25]. The 33 items on the CSHQ are grouped into eight subscales exploring a number of key sleep domains: (1) bedtime resistance (comprising six items); (2) sleep-onset delay (one item); (3) sleep duration (three items); (4) sleep anxiety (four items); (5) night waking (three items); (6) parasomnia (seven items); (7) sleep-disordered breathing (three items); and (8) daytime sleepiness (eight items). The total of 35 items (rather than 33) reflects the fact that two items of the bedtime resistance and sleep anxiety subscales are identical. Parents were asked to recall children’s recent sleep behavior during a typical week. Items were rated on a 3-point scale: *usually* if the particular sleep behavior occurred 5 to 7 times per week, *sometimes* if 2 to 4 times per week, and *rarely* if no or a single instance was recorded in a given week. Scores were adjusted to reflect the fact that a higher score was indicative of increased sleep disturbance.

#### Child morning-evening preference scale (CMEP)

Parents completed the CMEP, which is a 10-item multiple-choice scale adapted by Carskadon and colleagues [26] from the widely used Horne-Ostberg Morningness-Eveningness Questionnaire (MEQ) [37]. It has been used previously with school-aged children [38] and has shown good validity and reliability [38,39]. Scores on this measure range from 10 (indicating extreme evening preference) to 42 (indicating extreme morning preference). Therefore, higher scores on this measure indicate stronger morning preference, whereas lower scores indicate stronger evening tendencies. The CMEP demonstrates good validity and reliability [40] ranging between .83 and .95 with the MEQ [40], and a test-retest reliability coefficient of .78 [40].

#### Assessment of puberty

To monitor pubertal development in a non-intrusive manner, Petersen and colleagues (1988) developed a pubertal assessment interview (puberty development scale; PDS), which Carskadon and Acebo later devised a written version of [41]. The PDS asks adolescents to describe growth of auxiliary body hair, experience of a growth spurt, and skin changes [42]. For girls, additional items include menarche and breast changes. With boys, facial hair growth and voice deepening are explored. Development of each characteristic is rated on a 4-point scale ranging from 1 (*no development*) to 4 (*development complete*). The PDS exhibits adequate validity and reliability [41,43]. Higher scores on the PDS represent more mature pubertal development.

#### Analysis

Different demographics, physical, intellectual and psychiatric characteristics were subjected to between group comparisons using either one-way analysis of variance (ANOVA) or Chi-square analysis, depending on the nature of the data.

Sleep log information and CMEP scores were analyzed using one-way analyses of covariance (ANCOVA), with child age as a covariate and group as the between-subjects factor. Parental reports of sleep problems and PSG data were examined using multivariate analysis of covariance (MANCOVA). Group (*ADHD*, *Control*) was used as the between-subjects independent factor; sleep measures were dependent variables; and child age was a covariate.

Since we sought to consider the simultaneous influence of two variables (e.g., diagnostic status by circadian tendency or by externalizing behavior) on a third variable (e.g., sleep onset delay or bedtime resistance), and the interactions amongst variables, and since some of the independent variables were continuous, multiple regression analysis was employed. The CSHQ Sleep Onset Delay score, the CSHQ bedtime resistance score, and PSG-determined sleep onset latency were used as the outcome variables in the regression analyses.

Morningness/eveningness (reflecting circadian preference) and CBCL externalizing scores served as independent variables; effects of puberty and age were controlled for, as these variables are known to impact sleep latency. Following the recommendations of Aiken and West (1991), variables were entered into the regression equation in the following order: Simple model - mean age and pubertal status variables; Elaborated model - centered behavioral and circadian measures, and diagnostic status (with controls used as the reference) and, as the final step, the aforementioned interaction terms. Use of the interaction terms allowed us to explore whether ADHD status moderated predictor impact on outcomes.

SPSS Version 18.0 for Windows was employed in all statistical tests and a p value of <0.05 was considered to indicate statistical significance.

## Results

In Table 1, the frequencies, means, standard deviations, and F values of demographic and clinical characteristics of the children with ADHD and controls are shown separately for each group. Analyses of variance (ANOVAs) were performed to determine whether the groups differed in age, IQ, pubertal development or socioeconomic status. No significant between-group differences were observed for any of these measures. However, significant differences in clinical characteristics assessed using the CBCL were noted; children in the ADHD scored higher on Externalizing, Internalizing and Total Scores, and on the Conners’ Parents Rating Scale. Chi-square tests revealed no significant between-group differences in child gender or parental marital status.

**Table 1:** Demographic and clinical characteristics (Frequency or Mean and SD) of children with ADHD and controls

**Variable**	**ADHD (*n*** **= 26 )**	**Control (*n*** **= 49)**	**Test statistic**	***p***
Gender (M/F)	17/9	30/19	*χ*^2^(1) = 0.13	.72
Age	8.46 (*1.5*)	8.69 (*1.2*)	*F*(1,73) = 1.96	.17
Ethnic Background			*χ*^2^(4) = 4.31	.37
Caucasian	21	33		
African	0	1		
Asian	0	6		
Multi-ethnic	3	4		
Unknown	2	5		
SES	50.3 (*10.74*)	50.7 (*11.5*)	*F*(1,47) = .03	.86
IQ	98.9 (*17.5*)	105.7 (*13.6*)	*F*(1,68) = 2.34	.13
Pubertal Development	6.5 (*2.2*)	6.7 (1.7)	*F*(1,73) = .27	.61
Conners’ ADHD Index- parent	65.77 (*11.84*)	49.32 (7.64)	*F*(1,71) = 7.81	.007
ADHD subtype			*χ*^2^(3) = 58.67	<.001
No	0	49		
Combined	17	0		
Inattention	8	0		
Hyperactive-Impulsive	1	0		
Comorbid ODD	8	0	*χ*^2^(1) = 16.88	<.001
Comorbid CD	2	0	*χ*^2^(1) = 3.87	.05
CBCL				
Internalizing	60.13 (*8.71*)	50.42 (*9.02*)	*F*(1,73) = 19.7	<.001
Externalizing	59.2 (*9.25*)	49.7 (*8.52*)	*F*(1,73) = 24.1	<.001
Total	62.3 (*8.49*)	49.40 (9.04)	*F*(1,73) = 42.53	<.001

Note: ADHD = attention-deficit/hyperactivity disorder; SES = socioeconomic status; DISC-IV = Diagnostic Interview Schedule for Children, Fourth Edition; ODD = oppositional defiant disorder; CD = conduct disorder; CBCL = Child Behavior Checklist; All means are T-score transformed.

### Sleep measures, circadian preferences, and sleep disorders

Table 2 shows the means and standard deviations of sleep measures in children with ADHD and controls.

**Table 2:** Means and standard deviations for sleep measurements by group

**Measures**	**ADHD N = 26**	**Control N = 49**	***F***
Polysomnography			
Sleep latency	27.48 (17.99)	20.57 (16.24)	1.32
Total sleep time	519.20 (43.25)	518.84 (61.10)	0.09
Sleep efficiency (%)	94.91 (3.89)	96.90 (2.84)	3.62
Stage N1 (min)	27.50 (12.62)	22.55 (11.00)	1.47
Stage N2 (min)	204.36 (48.45)	185.17 (50.00)	1.29
Stage N3 (min)	201.73 (50.06)	224.01 (61.37)	1.82
REM Sleep (min)	85.61 (21.12)	87.11 (23.03)	0.47
PLM index	2.87 (1.82)	3.78 (1.24)	0.79
AHI Index	0.84 (0.97)	1.15 (0.61)	0.51
Sleep Logs			
Bedtime	21:13 (0:36)	21:14 (0:45)	0.30
Wake-up time	7:16 (0:45)	7:20 (0:53)	0.05
Sleep Duration	10:03	10:06	--
Morningness-Eveningness Scale	29.81 (4.56)	31.80 (3.32)	10.72*
Children’s Sleep Habits Questionnaire			
Bedtime Resistance	8.23 (2.60)	7.58 (2.43)	1.81
Sleep Onset Delay	1.71 (0.82)	1.42 (0.64)	3.98*
Sleep Duration	5.16 (1.93)	4.34 (1.45)	5.88*
Sleep Anxiety	6.10 (2.29)	5.23 (2.21)	4.06*
Night Wakings	4.58 (2.0)	3.73 (1.15)	6.88**
Parasomnias	9.06 (1.77)	8.27 (1.84)	3.57
Sleep Disordered Breathing	3.45 (0.93)	3.19 (0.90)	1.45
Daytime Sleepiness	13.71 (3.50)	11.81 (2.85)	8.07**
Total Sleep Disturbance Score	48.97 (5.82)	42.97 (7.16)	17.36**

Note. RLS refers to the Restless Leg Syndrome Index of the Pediatric Sleep Questionnaire; AHI refers to the apnea/hypopnea index. Children with primary sleep disorders were excluded from the study. *p ≤ .05. **p ≤ .001.

### Objective sleep measures

#### PSG measures

MANCOVAs conducted to explore between-group differences in PSG measures revealed no significant differences between groups.

### Subjective sleep measures

#### Sleep logs

MANCOVAs conducted to explore between-group differences in bedtime and wake-up time, as reported by sleep logs, revealed no significant differences between groups.

#### Child morning-evening preference (CMEP)

ANCOVAs evaluating between-group differences in reported sleep problems showed a significant main effect of group. Children with ADHD scored lower on this scale, indicating a stronger evening tendency compared to children in the control group.

#### Reported sleep problems

The MANCOVAs conducted to explore between-group differences in reported sleep problems on CSHQ measures revealed a significant main effect of group (*F* [1, 74] = 2.32; *p* < 0.05). Univariate analyses indicated that children with ADHD scored higher on the CSHQ subscales of Sleep Onset Delay, Sleep Duration, Night Waking, Sleep Anxiety, and Daytime Sleepiness. A separate ANCOVA evaluating between-group differences in Total CSHQ scores indicated that children with ADHD also scored higher on this measure.

### Predicting bedtime resistance and sleep onset latency

#### Correlations among outcome measures

To explore whether behavioral or circadian tendencies contributed to sleep onset problems in children with ADHD, multiple linear regression analyses were performed on data from 75 participants (Table 3). Inter-correlations between the variables used in the regression model and relevant outcome measures were conducted to examine possible colinearity confounds. Significant associations were found between parental report data on sleep-onset delay and PSG-measured sleep-onset latency (*r* = .58, *p* < .001), suggesting that these two measures, as expected, were examining similar constructs; CBCL externalizing scores were found to be negatively related to age (*r* = −.25, *p* < .05), such that younger children tended to have greater externalizing scores; no significant correlations were found between bedtime resistance and parental reports of sleep-onset delay or PSG-assessed sleep-onset latency or between any of the other predictor variables, suggesting colinearity assumptions were not violated.

**Table 3:** Results of regression analyses studying main effects and interaction effects of circadian and behavioral factors on the children’s sleep habits questionnaire controlling for age and pubertal development

**Measure**	**CSHQ bedtime resistance**			**CSHQ sleep onset delay**			**PSG sleep onset delay**		
	***B***	***SE B***	**β**	***B***	***SE B***	**β**	***B***	***SE B***	**β**
Control (Model 1)									
Age	−0.36	0.24	−0.18	0.05	0.07	0.10	0.49	1.97	0.03
PDS score	0.24	0.16	0.18	0.02	0.04	0.05	0.51	1.25	0.06
Total *R*^2^ (adjusted)	0.03			−0.01			−0.03		
Main effects (Model 2)									
Age	−0.18	0.24	−0.09	0.03	0.06	0.05	−0.91	1.88	−0.06
PDS score	0.18	0.16	0.13	−0.01	0.04	−0.03	0.30	1.14	0.03
Group (ADHD & Control)	−0.18	0.70	−0.03	0.01	0.18	0.01	4.23	5.14	0.11
MES for Children (Total)	−0.08	0.33	−0.03	−0.37**	0.08	−0.49	−8.16**	2.42	−0.43
CBCL: Externalizing (T score)	0.90*	0.34	0.35	0.03	0.09	0.04	−4.10	2.55	−0.22
Total *R*^2^ (adjusted)	0.10*			0.20**			0.16*		
Interaction (Model 3)									
Age	−0.12	0.24	−0.06	0.04	0.06	0.08	−0.93	1.93	−0.07
PDS score	0.16	0.16	0.12	−0.01	0.04	−0.02	0.70	1.21	0.08
Group (ADHD &Control)	−0.02	0.73	−0.00	0.09	0.19	0.06	5.46	5.42	0.15
MES for Children (Total Score)	−0.57	0.45	−0.21	−0.42**	0.12	−0.56	−5.80	3.33	−0.31
CBCL: Externalizing (T score)	1.03*	0.42	0.40	0.11	0.11	0.16	−2.62	2.90	−0.14
MES Total x Group	1.03	0.64	0.26	0.13	0.17	0.12	−4.63	5.11	−0.16
CBCL Externalizing x Group	−0.40	0.68	−0.10	−0.23	0.18	−0.21	−6.39	6.11	−0.16
Total *R*^2^ (adjusted)	0.11*			0.20*			0.16*		

*Note. B* and β refer to the unstandardized and standardized partial regression coefficients, respectively, and *SE B* refers to the standard error of the unstandardized coefficient. All group analyses use controls as the reference value. *R*2 values refer to the variation accounted for by the model. PDS refers to the Pubertal Development Scale; MES refers to the Morningness- Eveningness Scale; CBCL refers to the Child Behavior Checklist. In this model, the ADHD group is in reference to normal controls. *p ≤ .05. ** p ≤ .001.

#### Predicting parents’ reports of bedtime resistance

Regression analyses are reported in Table 3. Analyses revealed that externalizing problems contributed above and beyond puberty and age to prediction of parental reporting on bedtime resistance. No interaction effects of diagnostic group and predictors of outcome measures were evident and no other effects were significant.

#### Predicting parents’ reports of sleep onset delay

Analyses revealed that scores on the CMEP contributed above and beyond puberty and age in predicting parental report of sleep onset delay. No interaction effect between diagnostic group and any predictor of outcome measures was evident and no effect was significant, other than for CMEP scores (see Table 3).

#### Predicting PSG-measured sleep onset latency

Scores on the CMEP scale predicted PSG-measured sleep onset latency even when puberty and age were controlled for. Again, no interaction effect between diagnostic group and any predictor of outcome measures was evident and no effect was significant, other than for CMEP scores (Table 3).

## Discussion

The aim of the present study was to explore how behavioral (externalizing) and biological (circadian) factors previously found to be associated with ADHD symptoms contribute to sleep-onset insomnia and bedtime resistance commonly encountered in children with ADHD. In addition, we tested whether externalizing problems and circadian preferences differentially affect sleep problems in children with ADHD compared to controls. The results showed that each of the two factors (externalization and circadian preference) was associated with a different bedtime problem. In both groups of children, externalizing problems yielded significant independent contributions to parental reports of bedtime resistance, whereas an evening circadian tendency contributed both to parental reports of sleep onset delay and to PSG-measured sleep-onset latency. No significant interaction effect of behavioral/circadian tendency with ADHD status was evident.

Previous reports found that bedtime struggles and delayed sleep-onset were associated with sleep disorders characteristic of children with ADHD and suggested that what parents perceive to be behavioral problems might sometimes reflect the presence of circadian sleep disorders. Our present findings suggest that parents effectively differentiate between child “unwillingness” (bedtime refusal) versus “inability” (sleep-onset delay) to fall asleep. We further show that the “mixed bag” of bedtime problems in children with ADHD may have different etiologies.

Consistent with previous reports, we found that children with behavioral problems showed resistance at bedtime and considerable difficulty in settling down in the evening [13]. However, our findings suggest that children in the control group exhibit similar behaviors, and it was externalization *per se* that predicted parental descriptions of increased bedtime resistance and struggle. Previous reports have shown that sleep problems and externalizing behaviors often occur together [44,45]. Deficits in control of impulsive/aggressive behavior and emotions are likely to affect the ability of a child to settle down in the evening and the willingness to comply with parental requests to go to bed, resulting in increased bedtime resistance. Because such behaviors are characteristic of children with externalizing problems, not just children with ADHD, bedtime resistance might be better regarded as a general externalization problem rather than an aid in the diagnosis of ADHD. Additional studies are needed to increase our understanding of the associations between sleep and externalizing behavior and to further examine the biology of co-morbidity.

In the present study, we found that children with ADHD had a stronger circadian evening preference than did controls. This is in line with previous work showing that the evening increase in endogenous melatonin levels was delayed in medication-free children with ADHD [15]. Further, a stronger evening preference was predictive of both reported sleep-onset delay and PSG-measured sleep latency. Thus, circadian phase delay and bedtime refusal may be both common, but distinct, problems suggesting each may require a different interventional strategy.

### Additional findings

Consistent with the results of a number of other studies [13], we found that the extent of parent-reported sleep disturbance in children with ADHD was higher than that of healthy control subjects. More specifically, children with ADHD experienced significantly more sleep-onset problems than did controls and such children were more likely to be perceived as receiving an inadequate amount of nighttime sleep. In addition, such children had a higher level of reported daytime sleepiness, experienced more night waking, and suffered from higher sleep anxiety [46,47].

Although no significant between-group difference was found for bedtime resistance, it was noticeable that both groups of children showed levels of resistance previously considered to be characteristic of children with ADHD [5]. Hence, it may be that we have encountered a condition very prevalent in both controls and children with ADHD. Prevalence surveys based on parental reports identified bedtime resistance and night waking as the most common sleep problems in children [48-50]. Further, and consistent with the data of previous studies, although parental reports indicated higher levels of sleep problems in children with ADHD compared to controls, no significant between-group difference was evident when PSG was employed [51,52].

### Clinical implications

Our findings that bedtime problems associated with ADHD may vary in etiology emphasize the need for careful and accurate diagnosis of the source of sleep problems in children with ADHD. When assessing a child or adolescent with ADHD, it is necessary to distinguish a physiological sleep problem from a behavioral condition of which the sleep problem is but one of the symptoms.

To our knowledge, this is the first published study that used CMEP for the assessment of circadian tendencies in children with ADHD when compared with objective PSG measures of sleep. The findings of the present study emphasize the importance of using CMEP scale for the assessment of circadian preference in children with sleep onset problems, especially those with the diagnosis of ADHD.

Whereas PSG has been considered to be the “gold standard” for diagnosis of sleep disorders and in recording of sleep, PSG is both costly to implement and burdensome for families. Our present findings suggest that both objective and subjective measures could be used to provide complimentary information regarding potential sleep problems. Whereas parental reporting is a simple, reliable, and inexpensive method for accurately diagnosing sleep onset problems, PSG allows one to obtain objective, physiological information that can be used to examine primary sleep disorders that are common in children with inattention and hyperactivity. Given the high prevalence of ADHD and the elevated rates of associated sleep disorders, our finding that parental reporting is accurate is clinically important, as it can be used as the basis for an accurate and feasible assessment method in a clinical/pediatric setting. Pediatricians should be educated on this topic and provided with the scales for integration into practice.

### Limitations and future directions

Some limitations of this work are apparent. First, although the participants were below the cutoff score on the Pediatric Sleep Questionnaire, had no indication of desaturation, and no paradoxical breathing at the thoracic and abdominal channels, the presence of sleep-disordered breathing cannot be completely excluded because of the lack of nasal cannula or thermistor. Other limitations include the lack of employment of objective circadian measures, such as salivary melatonin level or core body temperature, to accurately determine the extent of the circadian discord. In addition, approximately one-third of the children with ADHD prescribed stimulants to control symptoms had potentially short wash-out periods. This should be noted, because a transient worsening of symptoms (a “rebound”) might have occurred after medication was stopped. Future studies should include a washout period of at least 1 week, or should examine only medication-naïve children when the impact of sleep practices on reported sleep problems is under consideration.

## Conclusion

In conclusion, our findings indicate that sleep onset problems in children with ADHD may be related to both behavioral factors and circadian preference. Our data further indicate that CSHQ is a useful clinical tool that should be incorporated into sleep evaluation in children with ADHD. Our findings add to the understanding of specific etiologies underlying sleep disorders and ADHD and contribute to closing the gap between data obtained using subjective or objective sleep measures in ADHD children.

## Competing interests

The authors declare that they have no competing interests.

## Authors’ contributions

RG has made substantial contributions to the conception and design of the study in addition to analysis and interpretation of data and writing the manuscript. LF, LB, SW have made substantial contributions to the acquisition of data and revision of the manuscript. AR has made substantial contributions to the analysis of data as a statistical consultant. SF has made substantial contributions to analysis of data. JC has made substantial contributions to the design of the study and interpretation of data. All authors have read and approved the final manuscript.

## Financial disclosure

This study was supported by grants to Dr. Gruber from the Canadian Institutes of Health Research (CIHR; grant number 153139) and the Fonds de la recherche en santé (FRSQ; grant number 10091). The authors of this manuscript declare no conflicts of interest.

## Pre-publication history

The pre-publication history for this paper can be accessed here:

http://www.biomedcentral.com/1471-244X/12/212/prepub
